# Post-heading dry-matter transport and nutrient uptake differentiate hybrid and inbred indica rice in the double-cropping system in South China

**DOI:** 10.3389/fpls.2024.1433402

**Published:** 2024-09-11

**Authors:** You-qiang Fu, Chu-sheng Lu, Xu-hua Zhong, Kai-ming Liang, Jun-feng Pan, Yan-zhuo Liu, Xiang-yu Hu, Rui Hu, Mei-Juan Li, Xin-yu Wang, Qun-huan Ye, Yuan-hong Yin, Ji-chuang Huang, Nong-rong Huang

**Affiliations:** ^1^ Rice Research Institute, Guangdong Academy of Agricultural Sciences/ Guangdong Key Laboratory of New Technology in Rice Breeding/ Key Laboratory of Genetics and Breeding of High Quality Rice in Southern China (Co-construction by Ministry and Province), Ministry of Agriculture and Rural Affairs, Guangzhou, China; ^2^ College of Natural Resources and Environment, South China Agricultural University, Guangzhou, China; ^3^ Institute of Agricultural Resources and Environment, Guangdong Academy of Agricultural Sciences, Guangzhou, China

**Keywords:** double-cropped rice, South China, inbred rice, hybrid rice, grain yield, nutrient absorption and utilization

## Abstract

**Introduction:**

Hybrid rice demonstrated superior performance in enhancing yield and efficiency in rice production compared to inbred rice. Nevertheless, the underlying mechanism responsible for the increased yield and efficiency of hybrid rice in South China’s double-cropping rice region remains understudied.

**Methods:**

Field experiments over two consecutive years were conducted. Firstly, yield variations among 20 inbred and 15 hybrid rice cultivars prevalent in South China’s double-cropping rice system were examined. Secondly, selecting representative hybrid and inbred rice cultivars with significant yield disparities were carried out on further analyzing dry-matter production, source-sink relationships, and nutrient absorption and utilization in both rice types.

**Results:**

Hybrid rice displayed an average grain yield of 8.07 and 7.22 t hm^-2^ in the early and late seasons, respectively, which corresponds to a 12.29% and 13.75% increase over inbred rice with statistically significant differences. In comparison to inbred rice, hybrid rice exhibited enhanced nitrogen concentration in leaves at the heading stage (15.48–16.20%), post-heading dry matter accumulation (52.62–73.21%), post-heading dry matter conversion rate (29.23–34.12%), and harvest index (17.31–18.37%). Additionally, grain nitrogen and phosphorus uptake in hybrid rice increased by 11.88–22.50% and 16.38–19.90%. Hybrid rice mainly improved post-heading nitrogen and phosphorus uptake and transport, while not total nitrogen and phosphorus uptake. Internal nitrogen and phosphorus use efficiency enhanced by 9.83%-14.31% and 10.15%-13.66%, respectively. Post-heading dry matter accumulation, harvest index, grain nitrogen and phosphorus uptake, and internal nitrogen and phosphorus use efficiency exhibited significant positive linear correlations with grain yield.

**Discussion:**

The period from heading to maturity is critical for enhancing hybrid rice yield and efficiency. Improving photosynthetic capacity during this period and promoting nutrient transport to grains serve as crucial pathways for increasing grain yield and efficiency. This study is of great significance for further improvement grain yield and breeding rice cultivars with high-yield and high nutrients use efficiency for South China's double-cropped rice system.

## Introduction

Rice sustains over half of the world's population as a crucial staple crop (Godfray et al., 2010). The two main subspecies cultivated in Asia, hybrid and inbred rice, displayed distinct differences in plant morphology, nutrient absorption, yield, quality, and stress tolerance (Wei et al., 2018; Chu et al., 2019; Liu et al., 2024). Mid-season hybrid indica rice has a higher reproductive organ biomass, while late-season hybrid indica rice showed the highest leaf biomass proportion (Chen et al., 2020a; Zhong et al., 2022). The hybrid indica rice yield is primarily associated with panicle and grain structure, whereas inbred japonica rice yield is mainly linked to productive panicle numbers (Liu et al., 2019). Compared to inbred rice cultivars, hybrid rice cultivars exhibited greater root oxidation activity and leaf photosynthetic rate, higher cytokinin content in the roots and leaves, and more remobilization of assimilates from the stem to the grain after heading stage (Zhou et al., 2023), implying obvious difference in the agronomic and physiological characteristics for hybrid and inbred rice cultivars. Hybrid rice cultivation in China has surpassed 50% of the total rice area (Peng, 2016). Compared to inbred rice, hybrid rice exhibited well-developed roots, robust tillering, and “double-high” properties, which refer to high grain yield and high nitrogen fertilizer use efficiency (Chen et al., 2020b; Zhu et al., 2020). Understanding the variation in agronomic characteristics for hybrid and inbred rice cultivars is essential for guiding high-yield and high-efficiency rice cultivation practices.

Hybrid rice, a product of crossbreeding two cultivars with complementary traits, exhibited heterosis, stress resilience, and improved yield potential compared to inbred rice (Wang et al., 2024). Hybrid rice is a landmark technological achievement in the technological innovation of rice production in China. Based on this technology, China is currently developing super hybrid rice with higher yield and high-quality. The increased yield and efficiency of hybrid rice are primarily attributed to enhanced biomass accumulation and efficient nutrient utilization (Qun et al., 2023). In the middle and lower reaches of the Yangtze River region, hybrid rice shows significantly superior grain yield, photosynthetic rate, leaf area index at the heading stage, dry matter accumulation at maturity stage, and nitrogen uptake compared to inbred rice (Huang et al., 2018; Pan et al., 2022). However, internal nitrogen use efficiency differences between cultivars are minimal, emphasizing the equal significance of biomass accumulation and nutrient uptake in hybrid rice yield (Chang et al., 2020; Liu et al., 2020). The according to Wei et al. (2018), compared to inbred indica or japonica rice, the improved nutrient absorption and utilization in indica-japonica hybrid rice can be attributed to its longer roots, greater root volume, and total active absorption surface area. Furthermore, Jiang et al. (2020) confirmed that hybrid rice enhances grain yield and nutrient uptake. The South China region is one of the main production areas of indica hybrid rice at low latitude in China, and the rice cultivation system here is traditionally characterized by double cropping rice. However, due to the influence of meteorological conditions such as overall higher temperature and smaller diurnal temperature difference during its whole growing season, the yielding merit of hybrid rice over the inbred varieties may not as strong as that in mountainous areas and/or slightly higher latitude area. Both hybrid and inbred rice types have traditionally been used for rice production in these areas. Hence, it is imperative to thoroughly investigate the advantages of hybrid rice in comparison to inbred rice cultivars in these regions. Theis will aid in formulating a robust strategy for future hybrid rice breeding and facilitate wide adoption of hybrid rice cultivars.

This study investigated grain yield differences and the mechanisms underlying dry-matter transport and nutrient use efficiency in both widely planting hybrid rice and inbred rice cultivars in South China. Purpose of this study is further improvement grain yield and breeding rice cultivars with high-yield and high-efficiency for South China’s double-cropped rice system.

## Materials and methods

### Plant materials

This study utilized 20 inbred and 15 hybrid rice cultivars that are extensively promoted in South China, with planting conducted during both early and late seasons in 2017. The more detail information for the rice cultivars was showed in **Table 1**.

**Table 1:** Basic information on 35 rice cultivars widely planted in South China’s double-cropping system.

Cultivars	Release number	Year	Region	Rice type
Yuejingsimiao2hao	Guangdong certified rice 2006067	2006	Guangdong	Inbred rice
Guangjingruanzhan	Guangdong certified rice 20170013	2017	Guangdong	Inbred rice
Fenghuazhan	Nation approved rice 2003037	2003	China	Inbred rice
Yuebiao5hao	Guangdong certified rice 2015031	2015	Guangdong	Inbred rice
Yueyousimiao	Guangdong certified rice 2011024	2011	Guangdong	Inbred rice
Yuemeizhan	Guangdong certified rice 20160034	2016	Guangdong	Inbred rice
Huanglizhan	Guangdong certified rice 2008001	2008	Guangdong	Inbred rice
Hefengyouzhan	Hainan certified rice 2014014	2014	Hainan	Inbred rice
Teqing	Guangdong certified rice 1988001	1988	Guangdong	Inbred rice
Yuejinyouzhan	Guangdong certified rice 2012024	2012	Guangdong	Inbred rice
Yuenongsimiao	Guangdong certified rice 2011023	2011	Guangdong	Inbred rice
Yuexiangzhan	Nation approved rice 20000005	2000	China	Inbred rice
Fengmeizhan	Nation approved rice 2006005	2006	China	Inbred rice
Hemeizhan	Guangdong certified rice 2008006	2008	Guangdong	Inbred rice
Huanghuazhan	Guangdong certified rice 2005010	2005	Guangdong	Inbred rice
Yuejinyinzhan	Guangdong certified rice 2015007	2015	Guangdong	Inbred rice
Helisizhan	Guangdong certified rice 2015033	2015	Guangdong	Inbred rice
Yinjingruanzhan	Guangdong certified rice 2006010	2006	Guangdong	Inbred rice
Yuehesimiao	Guangdong certified rice 2014026	2014	Guangdong	Inbred rice
Yuxiangyouzhan	Hainan certified rice 2007015	2007	Hainan	Inbred rice
Guang8you188	Guangdong certified rice 2012035	2012	Guangdong	Hybrid rice
Guang8you2168	Guangdong certified rice 2012007	2012	Guangdong	Hybrid rice
Shenyou9516	Guangdong certified rice 2010042	2010	Guangdong	Hybrid rice
Juliangyou751	Guangdong certified rice 2010041	2010	Guangdong	Hybrid rice
Tianyou998	Nation approved rice 2006052	2006	China	Hybrid rice
Teyou3301	Guangdong certified rice 2013018	2013	Guangdong	Hybrid rice
Yliangyou305	Guangdong certified rice 2016025	2016	Guangdong	Hybrid rice
Tianyouhuazhan	Nation approved rice 2012001	2012	China	Hybrid rice
Fengyousimiao	Guangdong certified rice 2003003	2003	Guangdong	Hybrid rice
Tianyou3618	Guangdong certified rice 2009004	2009	Guangdong	Hybrid rice
Wuyou303	Guangdong certified rice 20180020	2018	Guangdong	Hybrid rice
Wuyou308	Nation approved rice 2008014	2008	China	Hybrid rice
Tianyou3301	Nation approved rice 2010016	2010	China	Hybrid rice
Teyou524	Guangxi certified rice 1997004	1997	Guangxi	Hybrid rice
Yliangyou143	Guangdong certified rice 2012018	2012	Guangdong	Hybrid rice

For the 2018 early and late seasons, representative inbred and hybrid rice cultivars with comparable grain yield to mean yield of each rice type identified in 2017 were selected. The inbred rice cultivars chosen were Yuejinsimiao2hao, Huanghuazhan, and Yuehesimiao. The hybrid rice cultivars selected were Tianyouhuazhan, Juliangyou751, and Wuyou308.

### Soil physicochemical properties and climatic conditions

The experiments were conducted at the Baiyun Base of Guangdong Academy of Agricultural Sciences (113°23'E, 23°17'N, altitude 41.0 m) in Zhongluotan Town, Baiyun District, Guangzhou, Guangdong Province. The soil had a pH of 5.95, with organic matter at 22.48 g kg^-1^, total nitrogen at 1.29 g kg^-1^, total phosphorus at 0.42 g kg^-1^, total potassium at 8.43 g kg^-1^, alkali-hydrolyzable nitrogen at 58.03 mg kg^-1^, available phosphorus at 6.49 mg kg^-1^, and exchangeable potassium at 47.00 mg kg^-1^.

Standard ground meteorological stations monitored temperature, rainfall, and solar radiation post-rice sowing. During the rice growing season, the rainfall, average temperature, and total solar radiation were: 1461.40 mm, 24.06°, 1459.36 MJ·m^-2^ (early 2017 season), 342.20 mm, 27.26°, 1894.21 MJ·m^-2^ (late 2017 season); 910.8 mm, 24.65°, 1766.40 MJ·m^-2^ (early 2018 season), 705.20 mm, 25.68°, 1709.48 MJ·m^-2^ (late 2018 season).

### Experimental design

A randomized complete block design with three replications was used, with a planting spacing of 25.0 cm × 13.3 cm and a plot size of 16.8 m^2^. A government-recommended technology, namely “three controls” (Zhong et al., 2007), was employed in this study. Fertilization comprised 150 kg·hm^-2^ pure N, 45 kg·hm^-2^ P_2_O_5_, and 120 kg·hm^-2^ K_2_O, applied as urea, calcium superphosphate, and potassium chloride, respectively. The nitrogen fertilizer management ratio was 5:2:3 for basal, tillering, and panicle fertilizers, respectively. The basal fertilizer was applied one day before transplanting, tillering fertilizer 15 days after, and panicle fertilizer at panicle initiation. Phosphorus was applied entirely as the basal fertilizer, while potassium was split between the basal and panicle fertilizers. Diseases, pests and weeds were intensively controlled to avoid yield loss. In both 2017 and 2018, early season planting occurred on March 8th, transplanting on April 9th, and harvesting on July 11th. For the late season in 2017, planting took place on July 21st, transplanting on August 10th, and harvesting on November 6th. In 2018, late season planting, transplanting, and harvesting occurred on July 21st, August 8th, and November 19th, respectively.

### Measurement items and methods

#### Soil physicochemical properties

Soil physicochemical properties were determined as per soil agrichemical analysis methods (Bao, 2000).

#### Dry matter and yield

Rice plants were sampled at mid-tillering, panicle initiation, heading, and maturity stages, with 12 hills taken from each plot to count tillers per hill after removing their roots. At the heading stage, the stems, leaves, and panicles were separated, and leaf area was measured using an LI-3100 leaf area meter. During the maturity stage, the straw, filled grains, unfilled grains, and panicle rachises were separated and dried at 105°C for 20 min to stop enzymatic activity, followed by further drying at 75°C until a constant weight was reached, and then weighed. An automated seed counting system (TPZJ, MKY) was employed to count filled and unfilled grains, calculating grains per panicle, filled-grain percentage, and 1000-grain weight. Grain yield was measured from 150 hills at the maturity stage, and after drying to a constant weight at 105°C for 48 h, the moisture content was determined on 100 g of grain, and the final grain yield was adjusted to a 14% moisture content. The following calculations were:

Harvest index = grain weight / total biomass;

Grain weight/leaf-area ratio (mg·cm^-2^) = grain weight / leaf area at heading stage;

Post-heading dry matter accumulation (g·m^-2^) = dry weight at maturity - dry weight at heading stage;

Panicle dry matter accumulation (g·m^-2^) = dry weight of panicle at maturity - dry weight of panicle at heading stage;

Post-heading dry matter conversion rate (%) = post-heading dry matter accumulation / panicle dry matter accumulation × 100;

Stem-sheath (leaf) dry matter output (g·m^-2^) = stem-sheath (leaf) dry weight at heading stage - stem-sheath (leaf) dry weight at maturity;

Stem-sheath (leaf) dry matter output rate (%) = stem-sheath (leaf) dry matter output / stem-sheath (leaf) dry weight at heading stage × 100.

#### Determination of nitrogen and phosphorus content

Nitrogen and phosphorus content were determined by first grinding the samples and then digesting them using the H_2_SO_4_-H_2_O_2_ method. Subsequently, nitrogen was analyzed with an automatic nitrogen analyzer, while phosphorus was measured colorimetrically via the vanadium-molybdenum yellow method (Bao, 2000). The following calculations were performed:

Nitrogen concentration in leaves at heading stage (mg·kg^-1^) = nitrogen content in leaves at heading stage / leaf weight;

Nitrogen or phosphorus uptake in grains (g·m^-2^) = nitrogen or phosphorus concentration in grains × grain weight;

Nitrogen or phosphorus harvest index = nitrogen or phosphorus absorption in grains / total aboveground nitrogen or phosphorus accumulation at maturity;

Internal nitrogen or phosphorus use efficiency (kg·kg^-1^) = grain yield / total aboveground nitrogen or phosphorus accumulation at maturity;

Post-heading panicle nitrogen or phosphorus accumulation (g m^-2^) = nitrogen or phosphorus accumulation in panicles at maturity - nitrogen or phosphorus accumulation in panicles at heading stage;

Stem-sheath (leaf) nitrogen or phosphorus transport (g·m^-2^) = nitrogen or phosphorus accumulation in stem-sheath (leaf) at heading stage - nitrogen or phosphorus accumulation in stem-sheath (leaf) at maturity.

### Statistical analysis

**Figure 1:**
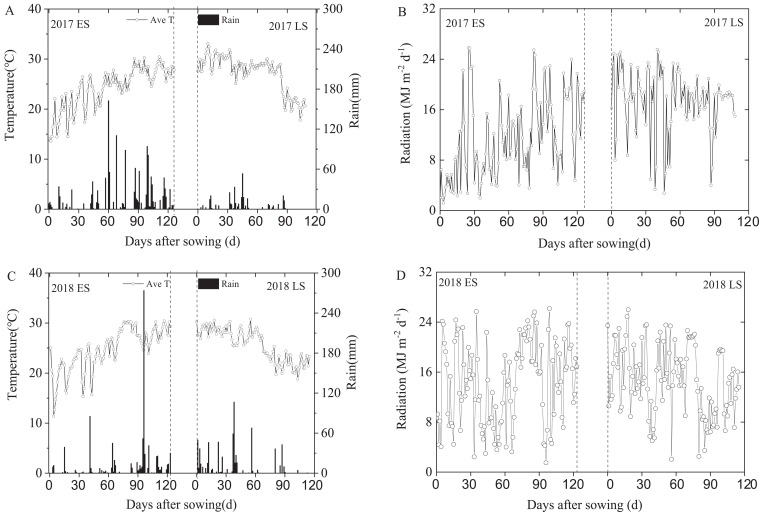
Daily mean temperature, precipitation **(A, C)** and solar radiation **(B, D)** during the entire rice growing season in Guangzhou City, Guangdong Province, China in 2017-2018. LS represents late-cropping season, ES represents early- cropping season. Ave T represents the average temperature.

The SPSS 13.0 software was utilized for data processing and analysis, while the OriginPro 9.0 software was employed for generating graphs. Statistically significant differences between treatments were denoted by different letters **Figure 1**.

## Results

### Yield and its determining factors

**Figure 2:**
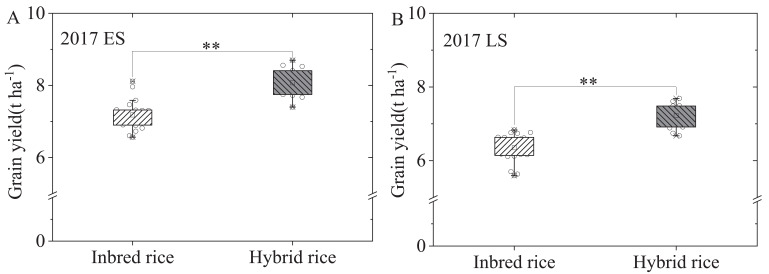
Grain yield of 20 inbred rice and 15 hybrid rice in South China in early **(A)** and late **(B)** seasons in 2017. LS represents late-cropping season, ES represents early- cropping season. ^**^ indicate significant differences at the 0.01 probability levels.

**Figure 2** illustrates the average rice grain yields for hybrid (15 cultivars) and inbred (20 cultivars) rice in the early and late seasons. Hybrid rice yields averaged for 8.07 t hm^-2^ and 7.22 t hm^-2^, representing 12.29% and 13.75% increases over inbred rice in the respective seasons, with statistically significant differences. No significant differences were observed in productive panicle number, spikelets per panicle, and filled-grain percentage between different rice cultivars in the early season. However, inbred rice showed significantly higher productive panicle number and spikelets per panicle in the late season compared to hybrid rice. Interesting, hybrid rice displayed a significantly higher (13.66%) filled-grain percentage than the inbred rice in the late season. Moreover, hybrid rice exhibited a significantly higher 1000-grain weight, with 15.18% and 13.61% increases in the early and late seasons, respectively, compared to inbred rice (**Table 2**; **Supplementary Table 1**).

**Table 2:** Grain yield and its components of inbred and hybrid rice in early and late seasons in 2018.

Season	Cultivar type	Cultivar	Panicle number (m^-2^)	Spikelet number (panicle^-1^)	Filled-grain percentage (%)	1000-grain weight(g)	Grain yield (t ha^-1^)
Early	Inbred rice	Yuejinsimiao2hao	280.13 a	195.84 a	70.42 b	20.34 b	6.41 b
		Huanghuazhan	285.26 ab	167.22 a	77.74 a	21.61 a	6.38 b
		Yuehesimiao	270.51 b	180.90 a	76.44 a	21.57 a	7.05 a
		Mean	278.63 A	181.36 A	74.72 A	21.14 B	6.61 B
	Hybrid rice	Tianyouhuazhan	280.13 a	181.49 a	77.68 a	24.87 a	8.02 a
		Juliangyou751	282.69 a	184.40 a	70.57 b	24.31 ab	7.32 b
		Wuyou308	295.51 a	210.16 a	78.12 a	23.87 b	8.21 a
		Mean	286.11 A	192.01 A	75.46 A	24.35 A	7.85 A
Late	Inbred rice	Yuejinsimiao2hao	266.67 a	181.11 a	69.97 b	21.38 b	6.04 a
		Huanghuazhan	266.67 a	186.92 a	72.10 b	21.19 b	5.47 b
		Yuehesimiao	258.34 a	181.50 a	81.02 a	22.34 a	6.47 a
		Mean	263.64 A	183.01 A	74.76 B	21.68 B	5.99 B
	Hybrid rice	Tianyouhuazhan	251.93 a	155.33 a	83.88 b	24.76 a	6.75 a
		Juliangyou751	249.36 a	157.00 a	80.52 c	24.88 a	7.00 a
		Wuyou308	257.69 a	161.44 a	90.51 a	24.31 b	6.67 a
		Mean	252.99 B	158.27 B	84.97 A	24.63 A	6.81 A
Analysis of variance							
Season(S)			**	**	**	*	**
Cultivar type(C)			NS	NS	**	**	**
S×C			*	**	**	NS	NS

Within the same column in each season, values (Mean ± standard error, n=3) followed by different letters have significant differences at the 0.05 probability level. NS stands for no significance; * and ** indicate significant differences at the 0.05 and 0.01 probability levels, respectively.

### Dry matter production

During the mid-tillering stage, hybrid rice exhibited 27.27% and 20.84% higher biomass than inbred rice in the early and late seasons, respectively. From mid-tillering to panicle initiation (MT-PI) period, both rice cultivars showed similar biomass accumulation in both seasons. The PI-HD period marked the highest biomass accumulation in rice, with inbred rice significantly outperforming hybrid rice in both seasons. However, during the HD-MA period, hybrid rice showed significantly increased biomass accumulation and harvest index, surpassing inbred rice by 73.21% and 18.37% in the early season, and 52.62% and 17.31% in the late season. Total biomass in early season hybrid rice was significantly higher than in inbred rice, while no significant difference was observed in the late season (**Table 3**).

**Table 3:** Biomass accumulation and harvest index of inbred and hybrid rice in early and late seasons in 2018.

Season	Cultivar type	Biomass accumulation during growth period(g m^-2^)				Total biomass (g m^-2^)	Harvest index
		MT	MT-PI	PI-HD	HD-MA		
Early	Inbred rice	34.62 b	208.50 a	794.41 a	296.25 b	1333.77 b	0.49 b
	Hybrid rice	44.06 a	219.55 a	668.79 b	513.14 a	1445.53 a	0.58 a
Late	Inbred rice	36.56 b	215.61 a	645.23 a	286.22 b	1160.04 a	0.52 b
	Hybrid rice	44.18 a	221.76 a	513.26 b	436.84 a	1216.04 a	0.61 a
Analysis of variance							
Season(S)		NS	NS	**	NS	**	**
Cultivar type(C)		**	NS	**	**	**	**
S×C		NS	NS	NS	NS	NS	NS

MT, PI, HD and MA represent Mid-tillering, Panicle initiation, Heading and Maturity stages, respectively. Within the same column in each season, values (Mean ± standard error, n=3) followed by different letters have significant differences at the 0.05 probability level. NS stands for no significance; * and ** indicate significant differences at the 0.05 and 0.01 probability levels, respectively.

### Dry matter accumulation and transfer

**Table 4** illustrates that hybrid rice exhibited a significant reduction in stem-sheath dry matter output, with a decrease of 15.02% during the early season and 13.32% during the late season, when compared to inbred rice. No significant differences were observed in the stem-sheath matter output rate between the two rice types. While compared to inbred rice, hybrid rice exhibited superior post-heading dry matter, conversion rate, and panicle dry matter, with increases of 73.21%, 34.12%, and 28.75%, respectively, during the early season and 52.62%, 29.23%, and 22.18%, respectively, during the late season.

**Table 4:** Biomass accumulation and transport of inbred and hybrid rice in early and late seasons in 2018.

Season	Cultivar type	Stem sheath dry matter output (g m^-2^)	Stem sheath dry matter output rate (%)	Post-heading dry matter accumulation (g m^-2^)	Post-heading dry matter conversion rate (%)	Panicle dry matter accumulation (g m^-2^)
Early	Inbred rice	300.99 a	28.77 a	296.25 b	49.44 b	597.23 b
	Hybrid rice	255.79 b	27.42 a	513.14 a	66.31 a	768.93 a
Late	Inbred rice	245.42 a	27.22 a	286.22 b	51.79 b	531.64 b
	Hybrid rice	212.74 b	26.98 a	436.84 a	66.93 a	649.58 a
Analysis of variance						
Season(S)		**	NS	NS	NS	**
Cultivar type(C)		**	NS	**	**	**
S×C		NS	NS	NS	NS	NS

Within the same column in each season, values (Mean ± standard error, n=3) followed by different letters have significant differences at the 0.05 probability level. NS stands for no significance; * and ** indicate significant differences at the 0.05 and 0.01 probability levels, respectively.

### Leaf nitrogen concentration and grain/leaf ratio

**Figure 3:**
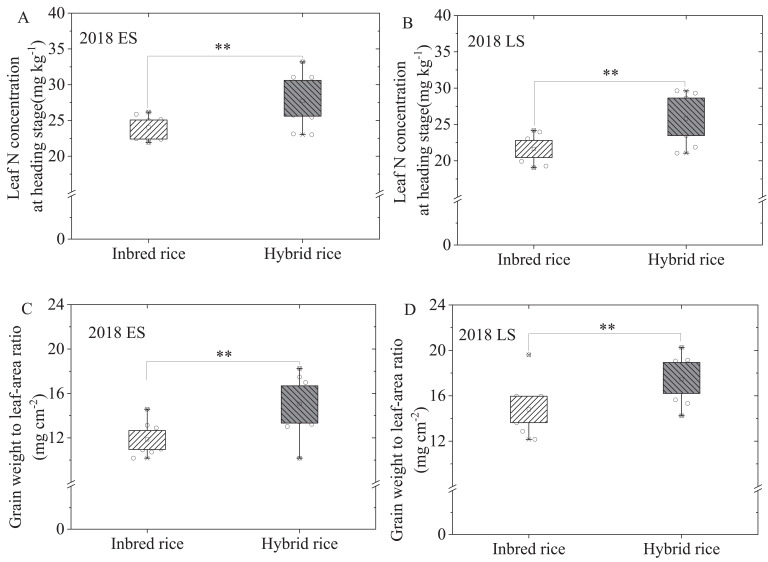
Leaf N concentration at heading stage **(A, B)** and grain weight to leaf-area ratio **(C, D)** of inbred and hybrid rice in early and late seasons in 2018. ^**^ indicate significant differences at the 0.01 probability levels.

**Figure 3** illustrates that hybrid rice had higher leaf nitrogen concentration at the heading stage than inbred rice increased by 15.48% (early season) and 16.20% (late season). Furthermore, the grain/leaf ratio of hybrid rice was significantly greater than that of inbred rice, with increases of 26.93% in early season and 18.01% in late season.

### Nitrogen uptake and utilization

**Table 5** indicated that nitrogen absorption in inbred and hybrid rice did not significantly differ at heading and maturity stages, including pre-heading stem-sheath nitrogen transport. However, hybrid rice exhibited greater post-heading panicle nitrogen accumulation, grain nitrogen absorption, nitrogen harvest index, and internal nitrogen use efficiency than inbred rice, with significant increases of 22.37%, 22.50%, 11.48%, and 9.83% in early season, and 11.74%, 11.88%, 10.77%, and 14.31% in late season(*P*<0.05).

**Table 5:** Nitrogen uptake and utilization of inbred and hybrid rice in early and late seasons in 2018.

Season	Cultivar type	N uptake at the HD (g m^-2^)	N uptake at the MA (g m^-2^)	Stem-sheath N transport (g m^-2^)	Post-heading panicle N accumulation (g m^-2^)	N uptake in grains (g m^-2^)	N harvest index	Internal N use efficiency (kg kg^-1^)
Early	Inbred rice	13.75a	11.76a	8.14 a	6.17 b	7.11 b	0.61 b	56.75 b
	Hybrid rice	13.82a	12.72a	8.78 a	7.55 a	8.71 a	0.68 a	62.33 a
Late	Inbred rice	9.46a	11.86a	4.22 a	6.56 b	7.66 b	0.65 b	50.04 b
	Hybrid rice	9.58a	11.96a	4.95 a	7.33 a	8.57 a	0.72 a	57.20 a
Analysis of variance								
Season(S)		**	NS	**	NS	NS	**	**
Cultivar type(C)		NS	NS	NS	**	**	**	**
S×C		NS	NS	NS	NS	NS	NS	NS

HD and MA represent Heading and Maturity stages, respectively. Within the same column in each season, values (Mean ± standard error, n=3) followed by different letters have significant differences at the 0.05 probability level. NS stands for no significance; * and ** indicate significant differences at the 0.05 and 0.01 probability levels, respectively.

### Phosphorus uptake and utilization

**Table 6** showed that, in the early season, inbred rice absorbed significantly more phosphorus at the flowering stage than hybrid rice, showing a 13.95% increase. However, at maturity, hybrid rice absorbed significantly more phosphorus than inbred rice, with an 8.13% increase. Compared to inbred rice, hybrid rice exhibited significant enhanced total phosphorus accumulation in panicles, grain phosphorus absorption, phosphorus harvest index, and internal phosphorus use efficiency by 19.65%, 19.90%, 11.94%, and 10.15%, respectively, in early season, and 15.13%, 16.38%, 16.67%, and 13.66% in late season(*P*<0.05).

**Table 6:** Phosphorus uptake and utilization of inbred and hybrid rice in early and late seasons in 2018.

Season	Cultivar type	P uptake at the HD (g m^-2^)	P uptake at the MA (g m^-2^)	Stem-sheath P transport (g m^-2^)	Post-heading panicle P accumulation (g m^-2^)	P uptake in grains (g m^-2^)	P harvest index	Internal P use efficiency (kg kg^-1^)
Early	Inbred rice	2.45 a	2.83 b	1.33 a	1.73 b	1.91 b	0.67 b	235.01 b
	Hybrid rice	2.15 b	3.06 a	1.16 b	2.07 a	2.29 a	0.75 a	258.86 a
Late	Inbred rice	2.10 a	2.67 a	0.95 b	1.52 b	1.77 b	0.66 b	227.24 b
	Hybrid rice	2.03 a	2.65 a	1.13 a	1.75 a	2.06 a	0.77 a	258.28 a
Analysis of variance								
Season(S)		**	**	**	**	**	NS	NS
Cultivar type(C)		**	NS	NS	**	**	**	**
S×C		*	NS	**	NS	NS	NS	NS

HD and MA represent Heading and Maturity stages, respectively. Within the same column in each season, values (Mean ± standard error, n=3) followed by different letters have significant differences at the 0.05 probability level. NS stands for no significance; * and ** indicate significant differences at the 0.05 and 0.01 probability levels, respectively.

### Correlation analysis

**Figure 4:**
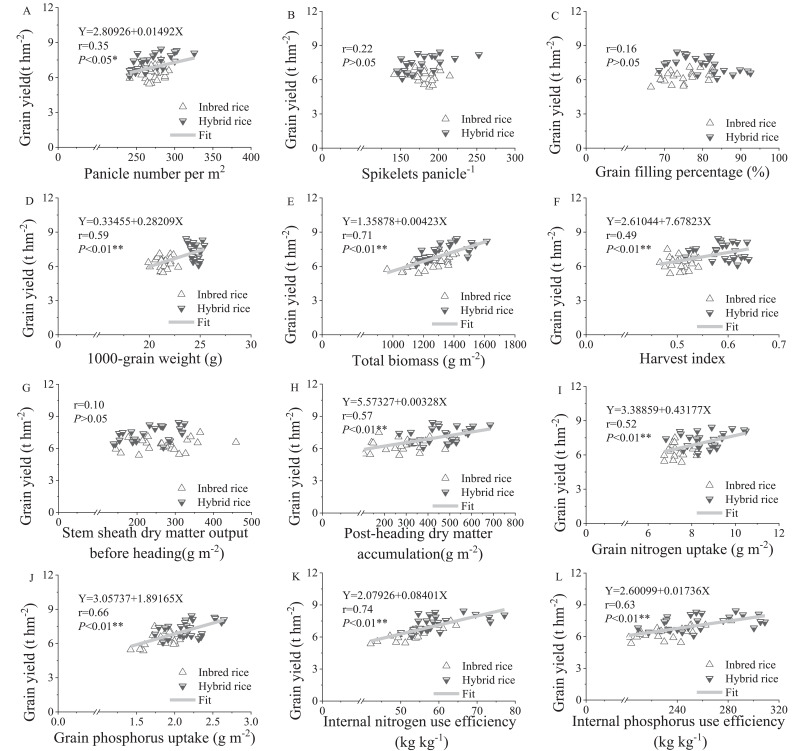
Correlations between grain yield and panicle number m^-2^ **(A)**, spikelets panicle^-1^ **(B)**, seed set rate **(C)**, 1000-grain weight **(D)**, total biomass **(E)**, harvest index **(F)**, stem sheath dry matter output of before heading **(G)**, post-heading dry matter accumulation **(H)**, grain nitrogen uptake **(I)**, grain phosphorus uptake **(J)**, internal nitrogen use efficiency **(K)** and internal phosphorus use efficiency **(L)** in the early and late seasons of 2018.

Significant or extremely significant linear positive correlations were observed between rice yield and the number of productive panicles (*P* < 0.05), thousand grain weight (*P* < 0.01), total biomass (*P* < 0.01), harvest index (*P* < 0.01), post-heading dry matter (*P* < 0.01), grain nitrogen uptake (*P* < 0.01), grain phosphorus uptake (*P* < 0.01), internal nitrogen use efficiency (*P* < 0.01), and internal phosphorus use efficiency (*P* < 0.01). In contrast, rice yield showed no significant correlation with the number of spikelets per panicle, filled-grain percentage, or pre-heading stem sheath dry matter output (**Figure 4**).

## Discussion

### Dry matter transport in hybrid and inbred rice cultivars

Numerous studies consistently demonstrated that hybrid rice can enhance rice yield by 10–20% compared to inbred rice (Huang, 2022; Liao et al., 2022; Meng et al., 2024). In a meta-analysis by Liao et al. (2022), hybrid rice demonstrated an 11% yield increase and an 8.1% increase in nitrogen uptake compared to inbred rice. Similarly, Xu et al. (2021) reported a 10.1% yield increase with hybrid rice over inbred rice. The current study's findings further bolster the productivity potential of hybrid rice, revealing a significant 12.29% rise in rice yields (early season) and 13.75% (late season) compared to inbred rice, with statistically significant differences (**Figure 2**). Grain yield, calculated as total biomass multiplied by the harvest index, benefits from several pathways in rice: increasing total biomass while maintaining the harvest index, enhancing the harvest index while sustaining total biomass, and simultaneously boosting both total biomass and harvest index. Numerous studies indicate that hybrid rice's increased yield stems from an extended growth period, leading to more effective accumulation of temperature and solar radiation, elevated net photosynthetic rate and carbon fixation, augmented leaf area index at the heading stage, and higher total biomass and grain yield (Xu et al., 2021; Pan et al., 2022). Moreover, studies suggest that indica-japonica hybrid rice features a larger leaf area index, improved panicle type, and a more rational canopy structure, resulting in enhanced post-heading light utilization and grain storage capacity (Chu et al., 2019; Zhou et al., 2023). This facilitates nutrient transport and accumulation, ultimately boosting the harvest index and rice yield (Fu et al., 2023). In the present study, both hybrid and inbred rice underwent simultaneous sowing, transplanting, and harvesting, managed using the three-control fertilization technique for rice. Consequently, under these experimental conditions, the primary factor driving increased production in hybrid rice was identified as elevated leaf nitrogen concentration at the heading stage (**Figure 3**), improved root development and canopy structure reasonably. This led to heightened accumulation and transportation of dry matter from post-heading towards the panicle (**Table 3**), thereby enhancing the harvest index and increasing rice yield (**Tables 2**, **3**), independent of an extended growth period or increased total biomass, in our study. This observation concurs with previous studies by Deng et al. (2023), which proposed that hybrid rice enhances net photosynthetic rate, cumulative CO_2_ fixation and the translocation of photosynthetic products to grains, thereby augmenting rice yield (Pan et al., 2022). However, the morphological and physiological mechanism underlying improving grain yield of hybrid rice cultivars in South China’s double-cropping rice system still needed further research.

Compared to inbred rice, hybrid rice demonstrates early and vigorous growth initially, leading to significantly higher biomass accumulation from transplanting to mid-tillering (**Table 3**), which is consistent with previous studies (Pan et al., 2020, 2023). However, there is no significant difference in biomass accumulation between hybrid and inbred rice from mid-tillering to panicle initiation. Subsequently, from panicle initiation to heading, hybrid rice showed significantly lower biomass accumulation than inbred rice. Nonetheless, from heading to maturity, hybrid rice displayed significantly higher biomass accumulation compared to inbred rice (**Table 3**). This contrasts with previous findings of significant differences in biomass accumulation in hybrid rice of indica-japonica variety reported in the middle and lower reaches of the Yangtze River or northern regions (Dong et al., 2020). The prevailing belief is that the early and vigorous growth advantage of hybrid rice in the initial stages persists, culminating in increased rice yield (Lu et al., 2021). The southern region, classified under the double-season rice ecological zone, faces limited light and temperature resources and shorter growth periods for each season. Increasing total biomass in inbred and hybrid under identical fertilization conditions is challenging. Consequently, hybrid rice primarily extends the duration of green leaf post-heading to enhance the accumulation and transport of post-heading biomass, thereby improving the harvest index and increasing rice yield (**Figure 3**, **Tables 2**, **4**). Moreover, this study employed the three-control fertilization technique for rice, which involves applying nitrogen fertilizer later in the growth cycle, resulting in relatively lower nitrogen application in the early stages. As hybrid rice exhibits early and vigorous growth, its nitrogen demand surpasses that of inbred rice, affecting biomass accumulation from mid-tillering to panicle initiation. Hence, there's no significant difference in biomass accumulation from mid-tillering to panicle initiation between hybrid and inbred rice (**Table 3**). With nitrogen fertilization shifted to panicle initiation to support rice panicle formation, hybrid rice develops a larger panicle structure compared to inbred rice, necessitating a longer panicle formation period. Consequently, biomass accumulation in hybrid rice is lower than in inbred rice from panicle initiation to heading (**Table 3**). Post-heading, owing to its larger storage capacity and higher leaf nitrogen content at the heading stage (**Figure 3**), hybrid rice enhances post-heading dry matter accumulation, the conversion rate of post-heading dry matter, panicle dry matter accumulation, the harvest index, and ultimately grain yield (**Tables 2**–**4**). Therefore, to improve the conversion rates of nutrient and dry matter after heading, moderate application of panicle fertilizer or heading fertilizer for hybrid rice cultivars is required to increase grain yield and nutrient use efficiency in South China’s double-cropping rice system.

### Nutrient absorption in hybrid rice and inbred rice

Xia et al. (2015) demonstrated that, in the middle and lower reaches of the Yangtze River, hybrid rice cultivars Liangyou Peijiu 9 and Yongyou 1 outperformed inbred cultivars Huanghuazhan and Yuxiangyouzhan in grain yield, mature biomass accumulation, nitrogen uptake, and nitrogen harvest index. Despite insignificant differences in internal nitrogen use efficiency between the cultivars, the superior performance of hybrid rice cultivars suggests a correlation between yield and nutrient absorption utilization, implied that the increase in grain yield is contributed to the enhanced the total nutrient uptake. Jiang et al. (2020) supported this finding, indicating that hybrid rice enhances yield by increasing the absorption of nitrogen, phosphorus, and potassium. Additionally, Meng et al. (2020a, b) also observed that hybrid indica-japonica rice cultivars Yongyou 2640 and Yongyou 1640 had significantly higher grain yield, nitrogen, and phosphorus accumulation at maturity than inbred japonica cultivars Yangjing 4038 and Yangjing 4227. Notably, these hybrid cultivars displayed higher nitrogen and phosphorus absorption in grains, but lower internal nitrogen and phosphorus use rates and post-heading leaf nitrogen and phosphorus transfer. Chen et al. (2018) demonstrated that in the middle and lower reaches of the Yangtze River, the indica-japonica hybrid rice cultivars Yongyou 12 and Jiayou Zhongke 6 exhibited greater rice yield, biomass, and nitrogen accumulation than inbred japonica cultivar Xiushui 134, though late-stage biomass and nitrogen transfer efficiency were lower in hybrid rice. Wei et al. (2018) revealed that indica-japonica hybrid rice cultivars have longer roots, the largest root volumes, and greater active absorption surface areas, leading to improved nitrogen, phosphorus, and potassium absorption and utilization. In South China’s double-cropping rice system, our study showed no significant difference in total nitrogen and phosphorus absorption between hybrid and inbred rice cultivars (**Tables 5**, **6**). However, hybrid rice exhibited significantly increased grain nitrogen and phosphorus uptake and internal nitrogen and phosphorus use efficiency (**Tables 5**, **6**), possibly due to the greater root architecture and the greener leaves area duration at heading stage for hybrid rice cultivars, with the synchronous increase in grain nitrogen and phosphorus uptake in hybrid rice being potentially linked to the genetic distance between the nitrogen and phosphorus absorption and utilization sites (Fu et al., 2019). Therefore, especially in South China’s double-cropping rice system, the application of panicle fertilizer is of great significance to improve the high-yield and nutrient use efficiency of hybrid rice, relative to inbred rice.

## Conclusions

In conclusion, hybrid rice exhibited significant yield advantages over inbred rice in the double-cropping rice region of southern China. This yield superiority is attributed to increased leaf nitrogen concentration during heading, enhanced biomass accumulation from heading to maturity, improved post-heading dry matter conversion rate, greater panicle biomass, and an elevated harvest index. The above results illustrate that the critical stage for increased yield and efficiency in hybrid rice lies in the period from heading to maturity. To further improve hybrid rice production and efficiency, focus should be given to enhancing photosynthetic capacity during the post-heading stage and facilitating nutrient transport to grains. Therefore, this study provides valuable theoretical support and a scientific foundation for the development of high-yield and high-efficiency breeding and cultivation techniques in rice.

## Data Availability

The original contributions presented in the study are included in the article/**Supplementary material**. Further inquiries can be directed to the corresponding author.

## Supplementary material

Supplementary Table 1 Grain yield and its components of 35 inbred and hybrid rice cultivars in early and late seasons in 2017. Within the same column in each season, values (Mean ± standard error, n=3) followed by different letters have significant differences at the 0.05 probability level. NS stands for no significance; * and ** indicate significant differences at the 0.05 and 0.01 probability levels, respectively.

## References

[B1] Bao S. D. (2000). Soil and agricultural chemistry analysis (Chinese Agriculture Press: Beijing).

[B2] Chang S. Chang T. Song Q. Wu J. Luo Y. Chen X.. (2020). Architectural and physiological features to gain high yield in an elite rice line YLY1. Rice 13, 1–16. doi: 10.1186/s12284-020-00419-y 32844350 PMC7447700

[B3] Chen M. Chen G. Di D. Kronzucker H. J. Shi W. (2020b). Higher nitrogen use efficiency (NUE) in hybrid ″super rice″ links to improved morphological and physiological traits in seedling roots. J. Plant Physiol. 251, 153191. doi: 10.1016/j.jplph.2020.153191 32585498

[B4] Chen G. Chen M. Zhang H. Wang S. Shi W. Cheng W. (2018). Differences of yield, accumulation and translocation properties of dry matter and n, and n use efficiency between indica-japonica hybrid rice and japonica rice. Acta Agricul Zhejiangensis 30, 1992–2000. doi: 10.3969/j.issn.1004-1524.2018.12.02

[B5] Chen S. Liu S. Yin M. Zheng X. Chu G. Xu C.. (2020a). Seasonal changes in crop growth and grain yield of different japonica rice cultivars in southeast China. Agron. J. 112, 215–227. doi: 10.1002/agj2.20049

[B6] Chu G. Chen S. Xu C. Wang D. Zhang X. (2019). Agronomic and physiological performance of indica/japonica hybrid rice cultivar under low nitrogen conditions. Field Crops Res. 243, 107625. doi: 10.1016/j.fcr.2019.107625

[B7] Deng J. Sheng T. Zhong X. Ye J. Wang C. Huang L.. (2023). Delayed leaf senescence improves radiation use efficiency and explains yield advantage of large panicle-type hybrid rice. Plants 12, 4063. doi: 10.3390/plants12234063 38068698 PMC10708015

[B8] Dong X. U. Ying Z. Chen Z. Chao H. Lei H. U. Shi Q.. (2020). Yield characteristics of japonica/indica hybrids rice in the middle and lower reaches of the Yangtze river in China. J. Integr. Agric. 19, 2394–2406. doi: 10.1016/S2095-3119(19)62872-8

[B9] Fu Y. Huang N. Zhong X. Mai G. Pan H. Xu H.. (2023). Improving grain yield and nitrogen use efficiency of direct-seeded rice with simplified and nitrogen-reduced practices under a double-cropping system in South China. J. Sci. Food Agric. 103, 5727–5737. doi: 10.1002/jsfa.12644 37076771

[B10] Fu Y. Zhong X. Pan J. Liang K. Liu Y. Peng B.. (2019). QTLs identification for nitrogen and phosphorus uptake-related traits using ultra-high density SNP linkage. Plant Sci. 288, 110209. doi: 10.1016/j.plantsci.2019.110209 31521212

[B11] Godfray H. C. J. Beddington J. R. Crute I. R. Haddad L. Lawrence D. Muir J. F.. (2010). Food security: the challenge of feeding 9 billion people. Science 327, 812–818. doi: 10.1126/science.1185383 20110467

[B12] Huang M. (2022). The decreasing area of hybrid rice production in China: causes and potential effects on Chinese rice self-sufficiency. Food Secur. 14, 267–272. doi: 10.1007/s12571-021-01199-z

[B13] Huang L. Sun F. Yuan S. Peng S. Wang F. (2018). Different mechanisms underlying the yield advantage of ordinary hybrid and super hybrid rice over inbred rice under low and moderate n input conditions. Field Crops Res. 216, 150–157. doi: 10.1016/j.fcr.2017.11.019

[B14] Jiang P. Xu F. Xiong H. Zhang L. Guo X. Zhu Y.. (2020). Integrated nitrogen management based on leaf diagnosis increases yield and fertilizer efficiency of direct-seeding rice. J. Plant Nutr. Fertilizers 26, 107–119. doi: 10.11674/zwyf.19032

[B15] Liao P. Meng Y. Weng W. Huang S. Zeng Y. Zhang H. (2022). Effects of hybrid rice on grain yield and nitrogen use efficiency: a meta-analysis. Scientia Agricul Sin. 55, 1546–1556. doi: 10.3864/j.issn.0578-1752.2022.08.006

[B16] Liu Q. Hu J. Zhou W. Yang Z. Chen Y. Ren W. (2019). Dry matter production and yield characteristics of machine-transplanted rice varieties falling into different types in Sichuan basin. Chin. J. Rice Sci. 33, 35–46. doi: 10.16819/j.1001-7216.2019.8049

[B17] Liu J. Wan J. Yin X. Gu X. Yin H. Yu M.. (2024). Progress and prospect of developing salt and alkali tolerant rice using hybrid rice technology in China. Plant Breed. 143, 86–95. doi: 10.1111/pbr.13115

[B18] Liu K. Yang R. Deng J. Huang L. Wei Z. Ma G.. (2020). High radiation use efficiency improves yield in the recently developed elite hybrid rice Y-Liangyou 900. Field Crops Res. 253, 107804. doi: 10.1016/j.fcr.2020.107804

[B19] Lu J. Liu K. Deng J. Feng X. Xiong X. Huang L.. (2021). Evaluating the effect of population density and the contribution of early canopy closure to grain yield of hybrid rice. J. Plant Growth Regul. 41, 1–10. doi: 10.1007/s00344-021-10342-1

[B20] Meng T. Ge J. Zhang X. Wei H. Liu Y. Li X.. (2020a). A dynamic model and its characteristics for nitrogen accumulation after transplanting in medium-maturity types of Yongyou japonica/indica hybrids. Acta Agronomica Sin. 46, 798–806. doi: 10.3724/SP.J.1006.2020.92046

[B21] Meng T. Ge J. Zhang X. Wei H. Zhou G. Dai Q. (2020b). Phosphorus accumulation characteristics of medium-maturity Yongyou japonica/indica hybrid rice after transplanting and its modeling. Chin. J. Rice Sci. 34, 256–265. doi: 10.16819/j.1001-7216.2020.9098

[B22] Meng X. Pan Y. Chai Y. Ji Y. Du H. Huang J.. (2024). Higher light utilization and assimilate translocation efficiency produced greater grain yield in super hybrid rice. Plant Soil, 1–16. doi: 10.1007/s11104-024-06639-1

[B23] Pan Y. Cao Y. Chai Y. Meng X. Wang M. Wang G.. (2023). Identification of photosynthetic parameters for superior yield of two super hybrid rice varieties: a cross-scale study from leaf to canopy. Front. Plant Sci. 14, 1110257. doi: 10.3389/fpls.2023.1110257 36866365 PMC9971572

[B24] Pan Y. Du H. Meng X. Guo S. (2022). Variation in photosynthetic induction between super hybrid rice and inbred super rice. Plant Physiol. Biochem. 178, 105–115. doi: 10.1016/j.plaphy.2022.03.005 35279007

[B25] Pan Y. Gao S. Xie K. Lu Z. Meng X. Wang S.. (2020). Higher radiation use efficiency produces greater biomass before heading and grain yield in super hybrid rice. Agronomy 10, 209. doi: 10.3390/agronomy10020209

[B26] Peng S. (2016). Dilemma and way-out of hybrid rice during the transition period in China. Acta Agronomica Sin. 42, 313–319. doi: 10.3724/SP.J.1006.2016.00313

[B27] Qun Z. Rui Y. Zhang W. Gu J. Liu L. Zhang H.. (2023). Grain yield, nitrogen use efficiency and physiological performance of indica/japonica hybrid rice in response to various nitrogen rates. J. Integr. Agric. 22, 63–79. doi: 10.1016/j.jia.2022.08.076

[B28] Wang C. Wang Z. Cai Y. Zhu Z. Yu D. Hong L.. (2024). A higher-yield hybrid rice is achieved by assimilating a dominant heterotic gene in inbred parental lines. Plant Biotechnol. J. 22, 1669–1680. doi: 10.1111/pbi.14295 38450899 PMC11123404

[B29] Wei H. Hu L. Zhu Y. Xu D. Zheng L. Chen Z.. (2018). Different characteristics of nutrient absorption and utilization between inbred japonica super rice and inter-sub-specific hybrid super rice. Field Crops Res. 218, 88–96. doi: 10.1016/j.fcr.2018.01.012

[B30] Xia B. Jiang P. Xie X. Zhao Y. Wei Y. Huang M.. (2015). Comparative study on yield formation and nutrient uptake and utilization between super hybrid rice and conventional rice. China Rice 21, 38–43. doi: 10.3969/j.issn.1006-8082.2015.04.007

[B31] Xu L. Yuan S. Wang X. Yu X. Peng S. (2021). High yields of hybrid rice do not require more nitrogen fertilizer than inbred rice: a meta-analysis. Food Energy Secur. 10, 341–350. doi: 10.1002/fes3.276

[B32] Zhong X. Huang N. R. Zheng H. Peng S. Buresh R. J. (2007). Specification for the ″three controls″ nutrient management technology for irrigated rice. Guangdong Agric. Sci. 5, 13–15. doi: 10.3969/j.issn.1004-874X.2007.05.003

[B33] Zhong X. Hussain H. A. Zhao B. Huang M. Hussain S. Xie R.. (2022). Analysis of grain yield formation components of extra heavy-panicle-type mid-season indica hybrid rice. J. Plant Growth Regul. 41, 1–18. doi: 10.1007/s00344-021-10353-y

[B34] Zhou Q. Yuan R. Zhang W. Gu J. Liu L. J. Zhang H.. (2023). Grain yield, nitrogen use efficiency and physiological performance of indica/japonica hybrid rice in response to nitrogen rates. J. Integr. Agric. 21, 2106–2118. doi: 10.1016/j.jia.2022.08.076

[B35] Zhu K. Zhou Q. Shen Y. Yan J. Xu Y. Wang Z.. (2020). Agronomic and physiological performance of an indica-japonica rice variety with a high yield and high nitrogen use efficiency. Crop Sci. 60, 1556–1568. doi: 10.1002/csc2.20150

