# Supplementary material for: Post-heading dry-matter transport and nutrient uptake differentiate hybrid and inbred indica rice in the double-cropping system in South China
Source: Front Plant Sci. 2024 Sep 11;15:1433402. doi: 10.3389/fpls.2024.1433402 (PMC11422115; doi:10.3389/fpls.2024.1433402)
Supplement: Supplementary Table 1 — Grain yield and its components of 35 inbred and hybrid rice cultivars in early and late seasons in 2017. Within the same column in each season, values (Mean ± standard error, n=3) followed by different letters have significant differences at the 0.05 probability level. NS stands for no significance; * and ** indicate significant differences at the 0.05 and 0.01 probability levels, respectively. [file Table1.docx]

**Supplementary data**

Table S1. Grain yield and its components of 35 inbred and hybrid rice cultivars in early and late seasons in 2017

| Season | Rice type | Rice cultivars | Panicle number (m^-2^) | Spikelet number (panicle^-1^) | Filled-grain percentage (%) | 1000-grain weight（g） | Grain yield (t ha^-1^) |
| --- | --- | --- | --- | --- | --- | --- | --- |
| Early season | Inbred rice | Helisizhan | 274.87 ab | 173.96 bc | 77.22 ab | 20.62 r | 7.46 d |
|  |  | Hemeizhan | 272.82 ab | 178.06 bc | 73.82 ab | 20.97 qr | 7.31 ef |
|  |  | Huanghuazhan | 246.16 d | 143.30 j | 75.13 ab | 24.79 e | 7.32 ef |
|  |  | Huanglizhan | 225.64 ef | 169.44 bc | 66.36 de | 24.3 f | 6.95 hi |
|  |  | Yuebiao5hao | 289.23 ab | 136.54 l | 76.93 ab | 22.65 l | 6.82 i |
|  |  | Yuehesimiao | 256.41 bc | 177.52 bc | 79.39 ab | 23.35 ij | 7.96 ab |
|  |  | Yuejingsimiao2hao | 229.74 ef | 183.79 ab | 69.55 bc | 22.09 n | 6.57 j |
|  |  | Yuejinyinzhan | 237.95 de | 170.75 bc | 76.67 ab | 22.76 k | 7.32 e |
|  |  | Yuejinyouzhan | 233.85 ef | 175.73 bc | 71.64 ab | 23.11 jk | 7.13 f |
|  |  | Yuemeizhan | 291.28 ab | 178.77 bc | 69.72 bc | 22.20 m | 6.91 hi |
|  |  | Yuenongsimiao | 274.87 ab | 148.46 ij | 77.25 ab | 23.49 i | 7.24 ef |
|  |  | Yuexiangzhan | 301.54 ab | 163.34 d | 79.06 ab | 20.18 s | 7.26 ef |
|  |  | Yueyousimiao | 291.28 ab | 151.34 g | 76.53 ab | 21.11 q | 6.89 hi |
|  |  | Yuxiangyouzhan | 211.28 fg | 193.5 ab | 80.66 a | 23.18 j | 8.12 ab |
|  |  | Fenghuazhan | 275.39 ab | 152.34 f | 76.05 ab | 21.38 pq | 6.97 h |
|  |  | Fengmeizhan | 276.93 ab | 154.39 f | 76.75 ab | 21.58 p | 7.11 f |
|  |  | Guangjingruanzhan | 262.56 b | 124.17 m | 76.5 ab | 24.51 f | 6.60 j |
|  |  | Hefengyouzhan | 213.34 f | 186.84 ab | 79.17 ab | 21.88 n | 7.05 g |
|  |  | Teqing | 272.82 ab | 140.17 k | 74.15ab | 25.07 cd | 7.12 f |
|  |  | Yinjingruanzhan | 244.11 de | 189.12 ab | 75.09 ab | 19.73 t | 7.58 c |
|  | Hybrid rice | Fengyousimiao | 299.49 ab | 147.02 ij | 67.44 c | 23.98 gh | 8.11 ab |
|  |  | Guang8you188 | 213.33 fg | 188.74 ab | 72.72 ab | 24.87 d | 7.40 de |
|  |  | Juliangyou751 | 320.00 a | 149.81 h | 72.26 ab | 24.59 ef | 7.75 b |
|  |  | Shenyou9516 | 242.05 de | 136.98 l | 77.42 ab | 28.60 b | 7.72 bc |
|  |  | Teyou524 | 205.13 g | 148.58 i | 81.05 a | 30.14 a | 8.56 ab |
|  |  | Yliangyou305 | 225.64 ef | 209.62 a | 74.53 ab | 21.76 o | 7.99 ab |
|  |  | Guang8you2168 | 244.11 de | 159.47 e | 69.14 bc | 25.51 cd | 7.67 bc |
|  |  | Teyou3301 | 227.69 ef | 154.01 f | 75.89 ab | 30.55 a | 7.97 ab |
|  |  | Tianyou3301 | 248.21 c | 166.76 c | 68.96 bc | 30.77 a | 8.53 ab |
|  |  | Tianyou3618 | 272.82 ab | 185.04 ab | 66.48 d | 23.63 hi | 8.16 ab |
|  |  | Tianyou998 | 254.36 bc | 178.92 bc | 62.49 e | 25.87 c | 7.85 ab |
|  |  | Tianyouhuazhan | 250.26 bc | 193.11 ab | 70.17 b | 24.55 ef | 8.05 ab |
|  |  | Wuyou303 | 248.21 c | 181.68 b | 72.64 ab | 24.04 g | 8.20 ab |
|  |  | Wuyou308 | 268.72 ab | 196.38 ab | 69.45 bc | 23.69 h | 8.32 ab |
|  |  | Yliangyou143 | 233.85 e | 166.27 cd | 71.76 ab | 27.86 b | 8.70 a |
|  |  |  |  |  |  |  |  |
| Late season | Inbred rice | Helisizhan | 258.46 cd | 172.28 ab | 85.78 ab | 19.68 mn | 6.37 ab |
|  |  | Hemeizhan | 272.82 b | 145.89 fg | 89.04 a | 19.96 m | 6.69 ab |
|  |  | Huanghuazhan | 260.51 cd | 145.12 fg | 85.41 ab | 25.21 d | 7.37 a |
|  |  | Huanglizhan | 260.52 cd | 149.73 ef | 89.49 a | 23.83 fg | 6.36 ab |
|  |  | Yuebiao5hao | 322.05 a | 130.71 i | 80.49 cd | 21.49 l | 6.55 ab |
|  |  | Yuehesimiao | 244.10 cd | 163.06 bc | 84.82 ab | 22.62 i | 6.66 ab |
|  |  | Yuejingsimiao2hao | 278.97 ab | 158.48 bc | 77.24 f | 21.56 k | 7.04 ab |
|  |  | Yuejinyinzhan | 281.03 ab | 144.95 fg | 83.02 bc | 22.30 j | 6.86 ab |
|  |  | Yuejinyouzhan | 233.85 cd | 163.49 b | 85.96 ab | 22.32 j | 5.74 b |
|  |  | Yuemeizhan | 262.56 cd | 144.60 fg | 80.54 c | 23.87 fg | 7.05 ab |
|  |  | Yuenongsimiao | 250.26 cd | 130.76 i | 86.27 ab | 23.07 h | 7.00 ab |
|  |  | Yuexiangzhan | 262.56 c | 152.08 d | 81.98 bc | 19.65 mn | 7.13 ab |
|  |  | Yueyousimiao | 270.77 bc | 149.25 f | 86.24 ab | 20.37 m | 6.99 ab |
|  |  | Yuxiangyouzhan | 237.95 cd | 178.79 ab | 83.08 bc | 22.22 jk | 7.05 ab |
|  |  | Fenghuazhan | 278.46 ab | 146.09 fg | 88.39 ab | 20.40 m | 6.79 ab |
|  |  | Fengmeizhan | 270.77 bc | 164.91 ab | 88.74 ab | 19.81 m | 6.56 ab |
|  |  | Guangjingruanzhan | 235.9 cd | 133.32 h | 89.62 a | 25.07 d | 6.66 ab |
|  |  | Hefengyouzhan | 260.52 cd | 164.53 a | 83.68 b | 21.37 l | 5.83 b |
|  |  | Teqing | 244.11 cd | 150.79 e | 77.87 e | 24.30 e | 7.20 ab |
|  |  | Yinjingruanzhan | 281.03 ab | 162.33 bc | 92.03 a | 19.01 n | 6.88 ab |
|  | Hybrid rice | Fengyousimiao | 311.79 ab | 139.16 g | 76.89 gh | 23.99 f | 7.22 ab |
|  |  | Guang8you188 | 242.05 cd | 175.28 ab | 87.61 ab | 24.99 de | 7.13 ab |
|  |  | Juliangyou751 | 258.46 cd | 150.66 ef | 80.01 de | 24.83 de | 6.67 ab |
|  |  | Shenyou9516 | 227.69 e | 146.74 fg | 88.20 ab | 29.33 b | 6.85 ab |
|  |  | Teyou524 | 209.23 f | 146.58 fg | 83.46 bc | 30.55 ab | 6.90 ab |
|  |  | Yliangyou305 | 229.75 d | 183.79 a | 89.08 a | 22.33 j | 7.45 a |
|  |  | Guang8you2168 | 264.62 bc | 145.16 fg | 83.67 bc | 25.35 d | 6.95 ab |
|  |  | Teyou3301 | 223.59 ef | 147.67 fg | 75.10 i | 30.55 a | 7.26 ab |
|  |  | Tianyou3301 | 240.00 cd | 146.66 fg | 75.36 h | 29.87 ab | 7.38 a |
|  |  | Tianyou3618 | 248.21 cd | 173.13 ab | 82.28 bc | 24.04 f | 6.50 ab |
|  |  | Tianyou998 | 270.77 bc | 156.35 c | 80.24 d | 25.20 d | 7.21 ab |
|  |  | Tianyouhuazhan | 268.72 bc | 161.20 bc | 77.00 g | 24.03 f | 7.54 a |
|  |  | Wuyou303 | 268.72 bc | 170.02 ab | 84.65 ab | 23.22 g | 6.35 ab |
|  |  | Wuyou308 | 281.02 ab | 162.64 bc | 82.60 bc | 23.58 fg | 6.86 ab |
|  |  | Yliangyou143 | 196.92 g | 172.78 ab | 82.67 bc | 27.96 c | 6.65 ab |
| Analysis of variance | | | | | | | |
| Season (S) | | | NS | ** | ** | NS | ** |
| Rice type (R) | | | * | * | ** | ** | ** |
| S×R | | | NS | NS | NS | NS | ** |

Note: Within the same column in each season, values (Mean±standard error, n=3) followed by different letters have significant differences at the 0.05 probability level. NS stands for no significance; * and ** indicate significant differences at the 0.05 and 0.01 probability levels, respectively.
